# C5a Increases the Injury to Primary Neurons Elicited by Fibrillar Amyloid Beta

**DOI:** 10.1177/1759091416687871

**Published:** 2017-01-01

**Authors:** Michael X. Hernandez, Pouya Namiranian, Eric Nguyen, Maria I. Fonseca, Andrea J. Tenner

**Affiliations:** 1 Department of Pathology and Laboratory Medicine, University of California, Irvine, School of Medicine, Irvine, USA; 2 Department of Molecular Biology and Biochemistry, University of California, Irvine, USA; 3 Department of Neurobiology and Behavior, University of California, Irvine, USA

**Keywords:** amyloid, C5a, C5aR1, complement, neurodegeneration, primary neurons

## Abstract

C5aR1, the proinflammatory receptor for C5a, is expressed in the central nervous system on microglia, endothelial cells, and neurons. Previous work demonstrated that the C5aR1 antagonist, PMX205, decreased amyloid pathology and suppressed cognitive deficits in two Alzheimer's Disease (AD) mouse models. However, the cellular mechanisms of this protection have not been definitively demonstrated. Here, primary cultured mouse neurons treated with exogenous C5a show reproducible loss of MAP-2 staining in a dose-dependent manner within 24 hr of treatment, indicative of injury to neurons. This injury is prevented by the C5aR1 antagonist PMX53, a close analog of PMX205. Furthermore, primary neurons derived from C5aR1 null mice exhibited no MAP-2 loss after exposure to the highest concentration of C5a tested. Primary mouse neurons treated with both 100 nM C5a and 5 µM fibrillar amyloid beta (fAβ), to model what occurs in the AD brain, showed increased MAP-2 loss relative to either C5a or fAβ alone. Blocking C5aR1 with PMX53 (100 nM) blocked the loss of MAP2 in these primary neurons to the level seen with fAβ alone. Similar experiments with primary neurons derived from C5aR1 null mice showed a loss of MAP-2 due to fAβ treatment. However, the addition of C5a to the cultures did not enhance the loss of MAP-2 and the addition of PMX53 to the cultures did not change the MAP-2 loss in response to fAβ. Thus, at least part of the beneficial effects of C5aR1 antagonist in AD mouse models may be due to protection of neurons from the toxic effects of C5a.

## Introduction

Alzheimer's disease (AD) is the most common form of dementia among the elderly. Characteristic neuropathological changes seen in AD brain include synaptic and neuronal loss, neurofibrillary tangles (NFTs), extracellular senile plaques composed of amyloid beta (Aβ) protein deposits, and evidence of inflammatory events. The relative contributions of these pathological markers to the cognitive dysfunction in AD remains controversial, but clinical and transgenic animal studies are increasingly suggesting that Aβ alone is not sufficient for the neuronal loss and subsequent cognitive decline observed in AD (Aizenstein et al., 2008; Blurton-Jones et al., 2009; Fonseca et al., 2009, and reviewed in Selkoe and Hardy, 2016). The role of neuroinflammation in neurodegenerative diseases, particularly relating to AD, is an active area of research (reviewed in (Wilcock et al., 2011; Wyss-Coray and Rogers, 2012)), and immune activation in the brain has been identified by many groups as a potential therapeutic target (Bachstetter et al., 2012, and reviewed in Heneka et al., 2015).

The complement system is a well-known part of the innate immune system that consists of more than 30 proteins found in plasma and cell surfaces, which protects from infection and aids in resolution of injury (Ricklin et al., 2010). Complement activation leads to the enhancement of phagocytosis of pathogens, apoptotic cells, and cellular debris as well as the generation of the anaphylatoxin C5a. This powerful anaphylatoxin can recruit phagocytes, such as microglia, to sites of infection or injury via its G-protein-coupled receptor C5aR1 (Yao et al., 1990; Nolte et al., 1996), as reviewed in (Klos et al., 2009). In the central nervous system, the C5aR1 receptor has been reported to be expressed by many cell types including microglia, astrocytes, and neurons (reviewed in Woodruff et al., 2010). While issues with antibody specificity and sensitivity have led to inconsistent reports, our lab has demonstrated specific C5aR1 staining on microglia that increases in AD mouse models (Ager et al., 2010).

Evidence is accumulating that most complement factors are synthesized in the brain upon insult and are upregulated in the brain in both humans and mouse models in aging and AD (Fonseca et al., 2011; Cribbs et al., 2012; Benoit et al., 2013). Moreover, the beta-sheet conformation of fibrillar amyloid beta (fAβ) has been shown to initiate the activation of the complement cascade by the classical and alternative pathways (reviewed in Alexander et al., 2008). Since the 1980s, complement factors have been known to be associated with fibrillar (thioflavine-staining) amyloid plaques found in AD patient brains (McGeer et al., 1989; Rogers et al., 1992; Afagh et al., 1996). In addition, plaques from mouse models of AD have also demonstrated extensive deposition of factors of complement (Akiyama et al., 2000; Matsuoka et al., 2001; Loeffler et al., 2008; Zhou et al., 2008).

Recent studies have shown that C5aR1 signaling is detrimental in various neurodegenerative disease mouse models (Woodruff et al., 2006; Woodruff et al., 2008; Fonseca et al., 2009; Brennan et al., 2015). Indeed, our previous study in two mouse models of AD showed a dramatic (50%–70%) decrease in amyloid plaque deposition, glial activation markers, and phosphorylated tau after 12 weeks of treatment with the C5aR1 antagonist PMX205. However, whether this protective effect was the result of an effect of the antagonist on microglia, where receptors have clearly been documented, or a direct effect on neurons or both could not be addressed in those models (Fonseca et al., 2009). The effects of C5a on neurons *in vitro* have been studied, although the results have varied greatly. Some studies have shown C5a can directly act on C5aR1 and cause apoptosis (Farkas et al., 1998; Pavlovski et al., 2012). Others have shown that addition of C5a can protect terminally differentiated neuroblastoma cells from Aβ toxicity (O'Barr et al., 2001). Given that cell lines do not always recapitulate findings in primary cells, we tested if C5a can enhance the injury to mouse primary neurons treated with fAβ *in vitro*.

Our new findings support previous results demonstrating that C5a can induce neuronal cell death. Both pharmacologic and genetic data indicate that C5aR1 is required for the C5a-induced decrease of neuronal MAP-2. Additionally, in the context of AD, we show that increased neuronal damage is caused by the addition of C5a to neurons treated with fAβ and can be blocked by the C5aR1 antagonist, PMX53. These findings suggest that at least part of the therapeutic benefit of C5aR1 antagonist seen earlier in mouse models of AD (Fonseca et al., 2009) may result from a direct protection of neurons and support further investigation of the use of such antagonists as part of a therapeutic strategy to slow the progression of AD and other neurological disorders in which complement activation may occur generating C5a in a neuronal environment in humans.

## Materials and Methods

### Reagents

Hanks' balanced salt solution (HBSS), serum-free neurobasal media (NB), B27 supplement (50x), L-glutamine, Prolong Gold anti-fade reagent with 4′,6-diamidino-2-phenylindole (DAPI), SuperScript III reverse transcriptase, SYBR/Green Master mix, and Alexa 405-, 488-, or 555-conjugated secondary antibodies were obtained from ThermoFisher. Poly-L-lysine hydrobromide, cytosine β-D-arabinofuranoside hydrochloride (Ara-C), and anti-actin antibody were from Sigma. Microtubule-associated protein 2 (MAP-2) antibody was obtained from Abcam. C5aR1 antibody (10/92) was obtained from SeroTec (Ager et al., 2010). Anti VGLUT1 and anti GAD67 antibodies were obtained from Millipore. HRP-conjugated F(ab')_2_ antibodies were from Jackson ImmunoResearch Laboratories. Human C5a peptide was obtained from CompTech. PMX53 was obtained from Cephalon. All buffers are made with Millipore-purified water with additional filters to eliminate LPS and are periodically tested for endotoxin using the *Limulus* amebocyte lysate clot assay (all solutions added to cells were <0.1 EU/mL; 1 EU is equivalent to 0.1 ng/mL LPS).

### β-Amyloid Synthesis, Purification, and Conformation Characterization

Human β-amyloid (1–42) (Aβ_1-42_), provided by Dr. Charles Glabe (University of California at Irvine), was synthesized by fluoren-9-ylmethoxy carbonyl chemistry using a continuous flow semiautomatic instrument as described previously (Burdick et al., 1992). The peptide was reconstituted in filter-sterilized water at a concentration of 1 mM after which an equal volume of 2 × TBS (0.033 M Tris, 0.267 M NaCl) was added (final concentration 500 µM Aβ). After 20 to 24 hr at 4 ℃ to allow fibril formation, aliquots were frozen for future use. The peptide conformation was analyzed by circular dichroism (CD) to confirm β-sheet conformation. Briefly, after using 1 × TBS as a blank, 200 µl of the peptide at 50 µM was run on a Jasco J-720 CD spectrometer and read from 200 nm to 250 nm with a step resolution of 0.5 nm and a scan speed of 20 nm/min. Four scans were acquired and averaged to generate the CD spectra of the peptide (Supplemental Figure S1; Li et al., 2004).

**Figure 1.**
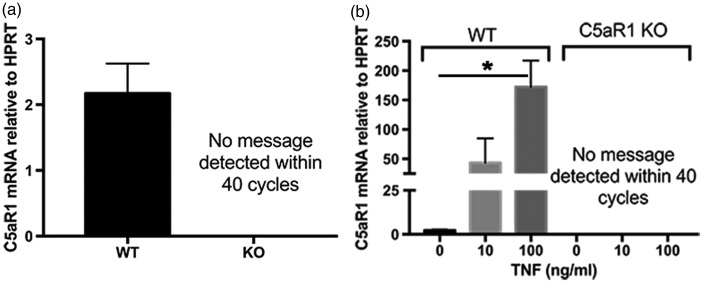
C5aR1 expression on primary neurons. Primary neurons from WT mice and C5aR1KO mice were generated using E15-E16 pups, cultured for 7 to 10 days, and RNA was collected from either (a) unstimulated cultures (WT *n* = 4; KO *n* = 2) or from (b) cultures treated with 0, 10, or 100 ng/ml of TNF-*α* for 24 hr (*n* = 2 for WT cells and *n* = 1 for C5aR1 knock out cells). Quantitative RT-PCR for C5aR1 expression relative to HPRT was performed with technical triplicates in each experiment. *p* values are calculated using unpaired two-tailed *t* test. Values of *p* < .05 were considered statistically significant.

### Animals, Neuron Isolation, and Culture

All animal experimental procedures were reviewed and approved by the Institutional Animal Care and Use Committee of University of California, Irvine. C57BL/6J were purchased from Jackson Laboratory and C5aR1 knock out (C5aR1KO) mice were originally a gift provided by Dr. Rick Wetsel (Hollmann et al., 2008). Pregnant mice were sacrificed by exposure to CO_2_ followed by cervical dislocation as a secondary method of euthanasia, after which the E15-E16 embryos were quickly removed, and the whole brains kept in Hank's balanced salt solution free of calcium and magnesium (CMF) and cleaned of meninges. Cerebral cortices were dissected out and exposed to 0.125% of trypsin in CMF for 7 min at 37 ℃. Cortical tissues were then resuspended in Dulbecco's modified eagle medium (DMEM) supplemented with fetal bovine serum (DMEM/FBS10%; endotoxin concentration ≤ 0.06 EU/mL) and dissociated by trituration using flame polished siliconized Pasteur pipettes. Viable cells, quantified by trypan blue exclusion, were plated at 1 to 2 × 10^5^ cells per well in 0.5 mL of DMEM/FBS10% on poly L-lysine (100 µg/mL) coated glass coverslips (Neuvitro) in 24-well plates (Costar, Cambridge, MA, USA). After 2 hr, the media was replaced with 0.5 mL serum-free neurobasal medium supplemented with B27 (NB/B27). On Day 3, the media was supplemented with Ara-C at 10 nM to reduce glia proliferation, and cultures were grown for 7 to 10 days *in vitro* before stimulation with 5 µM fAβ, human C5a (1, 10, and 100 nM; Benoit and Tenner, 2011) or PMX53 (100 nM). The concentration of fAβ chosen was previously shown to confer partial damage to primary rat neurons (Li et al., 2004) and confirmed with mouse neurons (Benoit et al., 2013). The dose of 100 nM C5a was selected for further interaction studies with fAβ after preliminary studies demonstrated 100 nM of C5a caused the biggest decrease in MAP-2 (Supplemental Figure S2). In some experiments, TNF-alpha was added on Day 7 at 0, 10, and 100 ng/mL for 24 hr, followed by lysis of neurons for RNA isolation for C5aR1 qRT-PCR.

### Immunocytochemistry

Neurons were fixed with 3.7% paraformaldehyde and permeabilized with 0.1% Triton X-100. Immunocytochemistry was performed as described previously (Benoit and Tenner, 2011). Briefly, after blocking with 10% FBS in PBS, neurons were incubated with chicken anti-MAP-2 antibody (dilution: 1:10,000) for 24 hr at 4 ℃. After three washes, coverslips were incubated with Alexa fluor 488-conjugated anti-chicken IgG antibody (dilution: 1:1,000) at room temperature (RT) for 1 hr. For colocalization experiments after blocking, cells were incubated with chicken anti MAP-2 antibody (9 μg/mL) and mouse anti GAD67 (1.5 μg/mL, clone 1G10.2, Millipore) or mouse anti VGLUT1 (1 μg/mL, Millipore) for 1 hr at RT. Cells were then incubated with the corresponding secondary antibodies, Alexa fluor 488-conjugated anti chicken IgG (dilution: 1:1,000) and Alexa 555 conjugated anti mouse IgG (dilution: 1:300) respectively for 1 hr at RT. The slides were mounted with 5 µL of Prolong Gold anti-fade reagent with DAPI. Cells were examined using the Axiovert 200 inverted microscope with AxioCam digital camera controlled by Axiovision program (Zeiss) or the Nikon Eclipse Ti-E fluorescent microscope and the NIS-Element AR 3.00 software. For each coverslip (three per condition, per experiment), three to five micrographs were taken to represent the entirety of the coverslip. The MAP-2 area was quantified using ImageJ software (Benoit et al., 2013). Briefly, the images were converted to grayscale, using the adjust “threshold” function under default conditions, the MAP-2 stained neurons were highlighted as regions of interest, and the area (as measured in pixels) was quantified using the “measure” function. The loss of MAP2 immunoreactivity has been demonstrated to be directly proportional to neuronal death (Brooke et al., 1999).

For colocalization of MAP-2 and GAD67 or VGLUT-1, images were analyzed using Zeiss Axiovision 4.6 software and percent of immunopositive area (% Field Area) (immunopositive area/total image area × 100) was determined. The mean % Field Area of each condition was obtained by averaging five to seven images per coverslip (three per condition).

### Western Blot

Neurons plated in 12-well plates at 2.5 × 10^5^ cells per well were washed with 1 mL Hank's Balanced Salt Solution (HBSS) and lysed with 200 µL of SDS reducing sample buffer. Samples were boiled for 5 min and equal amount of volume was loaded and separated by a 10% sodium dodecyl sulfate polyacrylamide gel electrophoresis (SDS-PAGE), transferred to nitrocellulose membrane (GE Healthcare), and then incubated with blocking buffer (5% powdered milk in Tris buffered saline (TBS)/Tween 0.1%) for 1 hr at RT. After washing, membranes were incubated overnight at 4 ℃ with rat anti-C5aR1 antibody (dilution: 1:1,000). After three washes, the membranes were incubated with HRP-conjugated anti-rat antibody (dilution: 1:5,000) for 1 hr at RT. The blots were developed using enhanced chemiluminescence plus (ECL+, GE Healthcare) and analyzed using the Nikon D700 digital SLR camera (Khoury et al., 2010) and ImageJ software. Equal protein loading for each lane per gel was confirmed by subsequent actin probing with mouse anti-actin antibody (dilution: 1:2,000) followed by HRP-conjugated anti-mouse antibody (dilution 1:5,000).

### RNA Extraction and qRT-PCR

Total RNA from cortical neuronal cultures was extracted using the Illustra RNAspin mini isolation kit (GE Healthcare). cDNA synthesis was performed using Superscript III reverse transcriptase following manufacturer's instructions. Quantitative RT-PCR was performed using the iCycler iQ and the iQ5 software (Bio-Rad) using SYBR/Green Master Mix. Mouse C5aR1 and HPRT primers were designed using primer-blast (ncbi.nlm.nih.gov) and obtained from Eurofins (Louisville, KY): C5aR1: Forward 5′-3′: GGGATGTTGCAGCCCTTATCA; Reverse 5′-3′: CGCCAGATTCAGAAACCAGATG. HPRT: Forward 5′-3′: AGCCTAAGATGAGCGCAAGT; Reverse 5′-3′: ATCAAAAGTCTGGGGACGCA. Quantitative RT-PCR data were only accepted if detected below 40 cycles of amplification.

### Statistical Analysis

All of the data are presented as mean ± SEM. Using Prism GraphPad, two-group comparisons were analyzed by the unpaired two-tailed Student's *t* test, and multiple-group comparisons were performed by one-way ANOVA uncorrected Fisher's LSD test as noted in Figure legends. Values of *p* < .05 were considered statistically significant. Each independent experiment consisted of a neuronal culture derived from its own unique litter of pups to attain biological replicates.

## Results

### Mouse Primary Neurons Express C5aR1

C5aR1 has previously been observed on a subset of neurons from both human and murine samples (O'Barr et al., 2001; Woodruff et al., 2010). To investigate whether the C5aR antagonist benefit seen in our previous *in vivo* AD mouse models could be in part due to a direct protective effect on neurons, primary mouse neuron cultures from E15.5 cortical neurons were cultured for 7 days and qRT-PCR was performed to determine C5aR1 expression in those primary mouse cultures. Unstimulated primary neurons showed C5aR1 mRNA in wild type primary neuron cultures but not neurons derived from C5aR1KO mice (Figure 1(a)). The expression of C5aR1 was upregulated by TNF-α in a dose-dependent manner (Figure 1(b)) within 24 hr of treatment in wild type but not C5aR1KO primary neurons. Protein expression in these neuronal cultures was below the level of detection with the proven specific anti C5aR1 antibody (10/92) by immunohistochemistry and by Western blot analysis. Anti-C5aR1 antibody reactivity was confirmed in primary neonatal microglia culture lysates derived from C5aR1-sufficient pups and which was absent in microglia from C5aR1KO pups (data not shown and Ager et al., 2010).

### C5a Is Toxic to Primary Neurons via C5aR1

**Figure 2.**
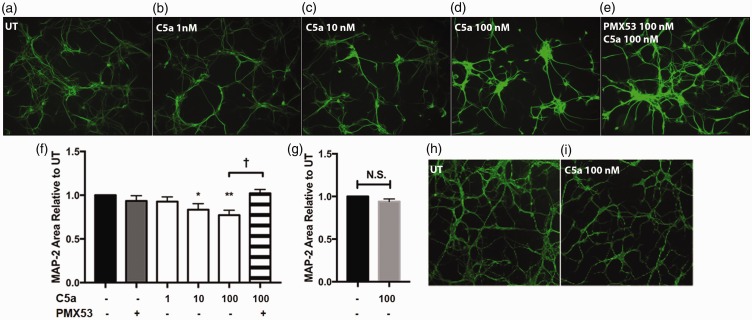
C5a causes MAP-2 loss in a dose-dependent manner. Primary neurons from WT mice (a–e and f) or C5aR1KO mice (h–i and g) were generated using E15-E16 pups and cultured for 7 to 10 days. The cells were then stimulated with the indicated concentrations of hC5a, or 100 nM PMX53 for 24 hr. MAP-2 was visualized by immunocytochemistry (10× magnification) and quantified (f–g) using ImageJ software as described in M & M. Data are presented as percentage of MAP-2 area relative to untreated (UT) ± *SEM. n* = 4 independent experiments (f) and *n* = 3 independent experiments (g), each experiment having three coverslips per treatment, three to five images per coverslip. *p* < .05 *, *p* < .01 ** relative to untreated cultures using one-way ANOVA, uncorrected Fisher's LSD test (F), and *p* < .02 † unpaired two-tailed *t* test (f, g).

Given that neuronal C5aR1 has been implicated in neurodegeneration and has been shown to lead to apoptosis of cultured neurons (Pavlovski et al., 2012), we tested whether purified human C5a was neurotoxic to our primary mouse cortical neurons. After 24 hr of exposure to C5a, the 10 nM and 100 nM concentrations of C5a tested were found to be damaging to neurons, as assayed by MAP-2 staining (a surrogate marker of neuronal health more sensitive than other assays, Brooke et al., 1999). The maximal concentration of 100 nM C5a led to the greatest MAP-2 loss (Figure 2(a)–(d), (f)). Pretreating the neurons with equal molar concentrations of the specific insurmountable C5aR1 antagonist, PMX53, completely prevented the MAP-2 loss induced by C5a (Figure 2(e)–(f)). To further confirm that C5a was acting through C5aR1 on neurons, primary neuron cultures were isolated from C5aR1KO mice, cultured and tested as with wild type neurons. Addition of C5a at 100 nM did not decrease MAP-2 compared with untreated C5aR1KO neurons (Figure 2(g)–(i)).

### C5a Enhances the Toxicity of Fibrillar Amyloid Beta

**Figure 3.**
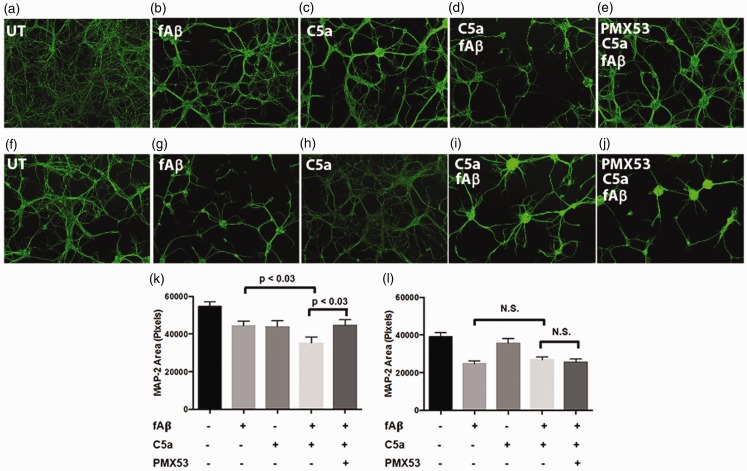
C5a increases toxicity in fAβ-treated neurons. Primary neurons from WT mice (a–e and k) or C5aR1KO mice (f–j and l) were generated using E15-E16 pups and cultured for 7 to 10 days. The cells were then stimulated or not with 5 µM fAβ, 100 nM hC5a, and 100 nM PMX53 for 24 hr. MAP-2 was visualized by immunocytochemistry (20 × magnification) and quantified using ImageJ software (k, l). Data are presented as mean MAP-2 area ± *SEM* of all image values from *n* = 3 independent experiments, each with three coverslips per treatment, five images per coverslip. *p* values are calculated using unpaired two-tailed *t* test. Values of *p* < .05 were considered statistically significant.

Complement components have been found to be upregulated in the AD brain, with many studies suggesting a detrimental role for complement in AD (Fonseca et al., 2009; Veerhuis, 2011). To determine whether C5a can further damage neurons treated with fAβ, 7- to 10-day primary neuronal cultures were exposed to fAβ that was verified to have beta-sheet structure by circular dichroism (Supplemental Figure S1). While 5 μM fAβ alone reduced MAP-2 by 18.5% within 24 hr (Figure 3(a)–(b)), addition of 100 nM C5a to fAβ-treated neurons further decreased MAP-2 by 20.8% relative to fAβ alone (Figure 3(d) and (k)). The additional decrease of MAP-2 observed after addition of C5a was blocked by pretreatment with PMX53 (Figure 3(e) and (k)). Neuron cultures derived from C5aR1KO mice did not show a decrease in MAP-2 relative to fAβ-treated neurons after C5a was added to the cultures (Figure 3(g)–(i), (l)). As expected, pretreatment with PMX53 did not protect C5aR1KO neurons from the fAβ-mediated toxicity (Figure 3(i), (j), and (l)).

### VGLUT1 and GAD67 Staining in Mouse Neuron Cultures

**Figure 4.**
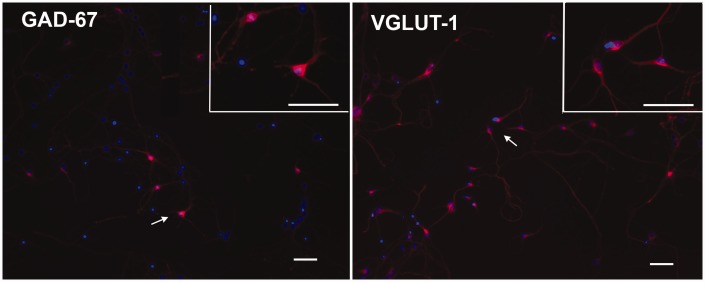
Primary neuronal cultures include GABAergic and glutamatergic neurons. Immunofluorescent staining of GAD67 (red, GABAergic neurons; left panel) or VGLUT1 (red, Glutamatergic neurons; right panel) in 7-day mouse neuron cultures. Nuclei are labeled with DAPI (blue; 10× magnification). Top right Inset in each panel is 20× magnification of region denoted by the arrow. Scale Bar: 50 µm.

To characterize the neuronal populations present in the 7- to 10-day mouse cultures and determine if either neuronal population was selectively affected by treatments, staining for GABAergic (GAD67+) and glutamatergic (VGLUT1+) markers was performed. The neuronal cultures were positive for both markers (Figure 4) and when treated with Aβ or C5a, no decrease in a selective population of neurons (glutamatergic or GABAergic) was seen (Supplemental Figure S3).

## Discussion

Our previous work demonstrated that blocking C5aR1 in two mouse models of AD resulted in a reduction of activated glia and amyloid deposits (Fonseca et al., 2009). Given the dominant expression of C5aR1 on microglia (Ager et al., 2010), it was hypothesized that the basis for the protective role of the C5aR1 antagonist *in vivo* was by inhibiting C5aR1-dependent proinflammatory responses of microglia. However, here we show that C5a leads to a decrease in MAP-2 staining when added to primary neuron cultures alone and increases neuronal damage resulting from Aβ treatment. PMX53, a C5aR1 specific antagonist (Otto et al., 2004), blocked the C5a-induced loss of MAP-2. Although C5aR1 protein in these neurons was not detectable using anti C5aR1 antibody (10/92), others have evidence of C5aR1 neuronal protein expression using a polyclonal chicken antibody (Pavlovski et al., 2012). Since primary neurons derived from C5aR1KO mice showed no C5a-induced loss of MAP-2, taken together these data provide evidence that neuronal C5aR1 is the receptor mediating the C5a induced neuronal damage and suggests that another beneficial consequence of C5aR1 antagonists in the context of AD is suppressing detrimental neuronal C5a-C5aR1 signaling.

The complement cascade is a powerful effector mechanism of the innate immune system that can be directly activated by fibrillar Aβ and has been implicated as a potential player in the inflammatory response in AD (reviewed in Wyss-Coray and Rogers, 2012). In brain, synthesis of most of the complement components increase during aging with a further increase in AD patients and animal models of AD consistent with a role for complement in the progression of the disease (Reichwald et al., 2009; Cribbs et al., 2012). Thus, as fibrillar plaques appear and complement component expression increase with age, complement will be activated generating C5a which will then diffuse and bind to C5aR1 expressing neurons and microglia.

Toll-like receptors (TLRs) are recognition components of the innate immune response that trigger protective inflammatory responses to the pathogens they recognize. TLR have been found to synergize with C5a-induced activation of phagocytes in the periphery (reviewed in Song, 2012; Hajishengallis et al., 2013) exacerbating a proinflammatory response thereby producing detrimental levels of proinflammatory cytokines (e.g., TNF-α, IL-1β). While neurons are also known to have TLR4 (Leow-Dyke et al., 2012), *in vitro*, we noted additive but not synergistic neuronal loss when C5a was added with fAβ to cultured neurons. While no preferential loss of GABAergic or glutamatergic neurons were seen in our cultures, it is possible that *in vivo* some subsets of neurons may be more susceptible to C5a mediated damage than others. Whether proinflammatory functions of C5a and TLR triggered in microglia may synergize to enhance neuronal dysfunction *in vivo* remains to be seen.

It is worth noting that addition of recombinant C5a has been shown to decrease cell viability in undifferentiated SH-SY5Y cells while retinoic acid (RA)-differentiated SH-SY5Y cells showed no change in viability to C5a and were protected against aggregated Aβ injury (O'Barr et al., 2001). These findings can be explained by the difference in biology when comparing cell lines used in that report to primary cultures used here or to the effect of RA-differentiation. More recent work using primary mouse neurons demonstrated recombinant C5a had a negative impact on neuronal viability (Pavlovski et al., 2012), which our study corroborates.

Primary neurons, although a better alternative to transformed and dividing cell lines, do not always recapitulate what happens *in vivo*. However, in our previous work with the 3xTg AD mouse model, PMX205, a close analog of PMX53, significantly and substantially reduced hyperphosphorylated tau by 69%. Thus, *in vivo* neurons were protected directly or indirectly when C5aR1 was inhibited. The *in vitro* data here thus suggest that the use of a C5aR1 antagonist could be beneficial in neurological diseases in which complement activation generating C5a occurs. PMX53 and PMX205 are cyclic hexapeptides based on the C-terminal end of C5a that differ only in substitution of hydrocinnamate for acetylated phenylalanine, (reviewed in Kohl, 2006), and are orally active, with PMX205 showing more favorable bioavailability (Woodruff et al., 2005). The C5aR1 specific antagonists PMX53 and/or PMX205 have been shown to reduce disease activity in several animal models, including models of central nervous system disease such as brain trauma (Sewell et al., 2004), amyotrophic lateral sclerosis (Woodruff et al., 2008) and Huntington-like neurodegeneration (Woodruff et al., 2006). In addition, the genetic deficiency of C5 has been shown to be one of a limited number of genetic differences associated with decreased amyloid deposition in DBA/2J mice versus C57Bl6 mice transgenic for the human APP gene (Ryman et al., 2008; Jackson et al., 2015), supporting a pathogenic role for C5a in the context of the AD brain *in vivo*.

When proposing systemic C5aR1 antagonist administration, it is important to consider the possibility that the recruitment of leukocytes into areas of infection may be dampened. However, the ability to generate bacteriolytic C5b-9 and to opsonize pathogens with C3b/iC3b (for ingestion and killing of pathogens) as well as the presence of antibody and T cell mechanisms of immune protection would remain intact. Thus, killing of C5b-9 susceptible microbes could still occur, as well as protective complement-independent and C3b/iC3b dependent antibody effector mechanisms (such as Fc receptor mediated phagocytosis, neutralization, complement opsonization and enhanced cytotoxicity). The lack of observable toxicity in multiple acute rodent models previously investigated and the extended treatment in our studies (Fonseca et al., 2009), lack of adverse effects in Phase 1 and 2 studies in humans of PMX53 (Kohl, 2006; Vergunst et al., 2007) and the current use of Eculizamab (anti C5) as a therapy in humans, suggest a promising safety profile of treatment with C5a receptor antagonists.

In summary, C5a was found to induce neuronal damage both alone and in the presence of amyloid β. This damage was prevented in the presence of PMX53 and absent for neurons genetically lacking C5aR1. These findings further implicate C5aR1 antagonists as a class of AD therapeutics that could have benefit in slowing progression of the disease while having limited negative suppressive effects on the immune system.

## Summary Statement

C5a and fibrillar amyloid beta together injure neurons greater than either protein alone. The additive damage to neurons can be blocked by the C5aR1 antagonist, PMX53. The data support further exploration of C5aR1 antagonists as therapy in Alzheimer's disease.

## Supplementary Material

Supplementary material

## References

[bibr1-1759091416687871] Afagh A. Cummings B. J. Cribbs D. H. Cotman C. W. Tenner A. J. (1996) Localization and cell association of C1q in Alzheimer's disease brain. Experimental Neurology 138: 22–32. 8593893 10.1006/exnr.1996.0043

[bibr2-1759091416687871] Ager R. R. Fonseca M. I. Chu S. H. Sanderson S. D. Taylor S. M. Woodruff T. M. Tenner A. J. (2010) Microglial C5aR (CD88) expression correlates with amyloid-beta deposition in murine models of Alzheimer's disease. Journal of Neurochemistry 113: 389–401. 20132482 10.1111/j.1471-4159.2010.06595.x PMC2921960

[bibr3-1759091416687871] Aizenstein H. J. Nebes R. D. Saxton J. A. Price J. C. Mathis C. A. Tsopelas N. D. Ziolko S. K. James J. A. Snitz B. E. Houck P. R. Bi W. Cohen A. D. Lopresti B. J. DeKosky S. T. Halligan E. M. Klunk W. E. (2008) Frequent amyloid deposition without significant cognitive impairment among the elderly. Archives of Neurology 65: 1509–1517. 19001171 10.1001/archneur.65.11.1509 PMC2636844

[bibr4-1759091416687871] Akiyama H. (2000) Inflammation and Alzheimer's disease. Neurobiology of Aging 21: 383–421. 10858586 10.1016/s0197-4580(00)00124-x PMC3887148

[bibr5-1759091416687871] Alexander J. J. Anderson A. J. Barnum S. R. Stevens B. Tenner A. J. (2008) The complement cascade: Yin-Yang in neuroinflammation-neuro-protection and -degeneration. Journal of Neurochemistry 107: 1169–1187. 18786171 10.1111/j.1471-4159.2008.05668.x PMC4038542

[bibr6-1759091416687871] Bachstetter A. D. Norris C. M. Sompol P. Wilcock D. M. Goulding D. Neltner J. H. St C. D. Watterson D. M. Van Eldik L. J. (2012) Early stage drug treatment that normalizes proinflammatory cytokine production attenuates synaptic dysfunction in a mouse model that exhibits age-dependent progression of Alzheimer's disease-related pathology. Journal of Neuroscience 32: 10201–10210. 22836255 10.1523/JNEUROSCI.1496-12.2012 PMC3419360

[bibr7-1759091416687871] Benoit M. E. Hernandez M. X. Dinh M. L. Benavente F. Vasquez O. Tenner A. J. (2013) C1q-induced LRP1B and GPR6 proteins expressed early in alzheimer disease mouse models, are essential for the C1q-mediated protection against amyloid-beta neurotoxicity. Journal of Biological Chemistry 288: 654–665. 23150673 10.1074/jbc.M112.400168 PMC3537064

[bibr8-1759091416687871] Benoit M. E. Tenner A. J. (2011) Complement protein C1q-mediated neuroprotection is correlated with regulation of neuronal gene and microRNA expression. Journal of Neuroscience 31: 3459–3469. 21368058 10.1523/JNEUROSCI.3932-10.2011 PMC3080046

[bibr9-1759091416687871] Blurton-Jones M. Kitazawa M. Martinez-Coria H. Castello N. A. Muller F. J. Loring J. F. Yamasaki T. R. Poon W. W. Green K. N. LaFerla F. M. (2009) Neural stem cells improve cognition via BDNF in a transgenic model of Alzheimer disease. Proceedings of the National Academy of Sciences of the United States of America 106: 13594–13599. 19633196 10.1073/pnas.0901402106 PMC2715325

[bibr10-1759091416687871] Brennan F. H. Gordon R. Lao H. W. Biggins P. J. Taylor S. M. Franklin R. J. Woodruff T. M. Ruitenberg M. J. (2015) The complement receptor C5aR controls acute inflammation and astrogliosis following spinal cord injury. Journal of Neuroscience 35: 6517–6531. 25904802 10.1523/JNEUROSCI.5218-14.2015 PMC6605214

[bibr11-1759091416687871] Brooke S. M. Bliss T. M. Franklin L. R. Sapolsky R. M. (1999) Quantification of neuron survival in monolayer cultures using an enzyme-linked immunosorbent assay approach, rather than by cell counting. Neuroscience Letters 267: 21–24. 10400239 10.1016/s0304-3940(99)00315-8

[bibr12-1759091416687871] Burdick D. Soreghan B. Kwon M. Kosmoski J. Knauer M. Henschen A. Yates J. Cotman C. Glabe C. (1992) Assembly and aggregation properties of synthetic Alzheimer's A4/beta amyloid peptide analogs. Journal of Biological Chemistry 267: 546–554. 1730616

[bibr13-1759091416687871] Cribbs D. H. Berchtold N. C. Perreau V. Coleman P. D. Rogers J. Tenner A. J. Cotman C. W. (2012) Extensive innate immune gene activation accompanies brain aging, increasing vulnerability to cognitive decline and neurodegeneration: A microarray study. Journal of Neuroinflammation 9: 179. 22824372 10.1186/1742-2094-9-179 PMC3419089

[bibr14-1759091416687871] Farkas I. Baranyi L. Takahashi M. Fukuda A. Liposits Z. Yamamoto T. Okada H. (1998) A neuronal C5a receptor and an associated apoptotic signal transduction pathway. Journal of Physiology 507(Pt 3): 679–687. 9508829 10.1111/j.1469-7793.1998.679bs.x PMC2230831

[bibr15-1759091416687871] Fonseca M. I. Ager R. R. Chu S. H. Yazan O. Sanderson S. D. LaFerla F. M. Taylor S. M. Woodruff T. M. Tenner A. J. (2009) Treatment with a C5aR antagonist decreases pathology and enhances behavioral performance in murine models of Alzheimer's disease. Journal of Immunology 183: 1375–1383. 10.4049/jimmunol.0901005 PMC4067320 19561098

[bibr16-1759091416687871] Fonseca M. I. Chu S. H. Berci A. M. Benoit M. E. Peters D. G. Kimura Y. Tenner A. J. (2011) Contribution of complement activation pathways to neuropathology differs among mouse models of Alzheimer's disease. Journal of Neuroinflammation 8: 4. 21235806 10.1186/1742-2094-8-4 PMC3033336

[bibr17-1759091416687871] Hajishengallis G. Abe T. Maekawa T. Hajishengallis E. Lambris J. D. (2013) Role of complement in host-microbe homeostasis of the periodontium. Seminars in Immunology 25: 65–72. 23684627 10.1016/j.smim.2013.04.004 PMC3706506

[bibr18-1759091416687871] Harvey H. Durant S. (2014) The role of glial cells and the complement system in retinal diseases and Alzheimer's disease: Common neural degeneration mechanisms. Experimental Brain Research 232: 3363–3377. 25183160 10.1007/s00221-014-4078-7

[bibr19-1759091416687871] Heneka M. T. (2015) Neuroinflammation in Alzheimer's disease. Lancet Neurology 14: 388–405. 25792098 10.1016/S1474-4422(15)70016-5 PMC5909703

[bibr20-1759091416687871] Hollmann T. J. Mueller-Ortiz S. L. Braun M. C. Wetsel R. A. (2008) Disruption of the C5a receptor gene increases resistance to acute Gram-negative bacteremia and endotoxic shock: Opposing roles of C3a and C5a. Molecular Immunology 45: 1907–1915. 18063050 10.1016/j.molimm.2007.10.037 PMC4294580

[bibr21-1759091416687871] Jackson H. M. Onos K. D. Pepper K. W. Graham L. C. Akeson E. C. Byers C. Reinholdt L. G. Frankel W. N. Howell G. R. (2015) DBA/2J genetic background exacerbates spontaneous lethal seizures but lessens amyloid deposition in a mouse model of Alzheimer's disease. PLoS ONE 10: e0125897. 25933409 10.1371/journal.pone.0125897 PMC4416920

[bibr22-1759091416687871] Khoury M. K. Parker I. Aswad D. W. (2010) Acquisition of chemiluminescent signals from immunoblots with a digital single-lens reflex camera. Analytical Biochemistry 397: 129–131. 19788886 10.1016/j.ab.2009.09.041 PMC2808431

[bibr23-1759091416687871] Klos A. Tenner A. J. Johswich K. O. Ager R. R. Reis E. S. Kohl J. (2009) The role of the anaphylatoxins in health and disease. Molecular Immunology 46: 2753–2766. 19477527 10.1016/j.molimm.2009.04.027 PMC2725201

[bibr24-1759091416687871] Kohl J. (2006) Drug evaluation: The C5a receptor antagonist PMX-53. Current Opinion in Molecular Therapeutics 8: 529–538. 17243489

[bibr25-1759091416687871] Leow-Dyke S. Allen C. Denes A. Nilsson O. Maysami S. Bowie A. G. Rothwell N. J. Pinteaux E. (2012) Neuronal Toll-like receptor 4 signaling induces brain endothelial activation and neutrophil transmigration in vitro. Journal of Neuroinflammation 9: 230. 23034047 10.1186/1742-2094-9-230 PMC3481358

[bibr26-1759091416687871] Li M. Pisalyaput K. Galvan M. Tenner A. J. (2004) Macrophage colony stimulatory factor and interferon-gamma trigger distinct mechanisms for augmentation of beta-amyloid-induced microglia-mediated neurotoxicity. Journal of Neurochemistry 91: 623–633. 15485493 10.1111/j.1471-4159.2004.02765.x

[bibr27-1759091416687871] Loeffler D. A. Camp D. M. Bennett D. A. (2008) Plaque complement activation and cognitive loss in Alzheimer's disease. Journal of Neuroinflammation 5: 9. 18334032 10.1186/1742-2094-5-9 PMC2292690

[bibr28-1759091416687871] Matsuoka Y. Picciano M. Malester B. LaFrancois J. Zehr C. Daeschner J. M. Olschowka J. A. Fonseca M. I. O'Banion M. K. Tenner A. J. Lemere C. A. Duff K. (2001) Inflammatory responses to amyloidosis in a transgenic mouse model of Alzheimer's disease. American Journal of Pathology 158: 1345–1354. 11290552 10.1016/S0002-9440(10)64085-0 PMC1891893

[bibr29-1759091416687871] McGeer P. L. Akiyama H. Itagaki S. McGeer E. G. (1989) Activation of the classical complement pathway in brain tissue of Alzheimer patients. Neuroscience Letters 107: 341–346. 2559373 10.1016/0304-3940(89)90843-4

[bibr30-1759091416687871] Nolte C. Möller T. Walter T. Kettenmann H. (1996) Complement 5a controls motility of murine microglial cells *in vitro* via activation of an inhibitory G-protein and the rearrangement of the actin cytoskeleton. Neuroscience 73: 1091–1107. 8809827 10.1016/0306-4522(96)00106-6

[bibr31-1759091416687871] O'Barr S. A. Caguioa J. Gruol D. Perkins G. Ember J. A. Hugli T. Cooper N. R. (2001) Neuronal expression of a functional receptor for the C5a complement activation fragment. Journal of Immunology 166: 4154–4162. 10.4049/jimmunol.166.6.4154 11238666

[bibr32-1759091416687871] Otto M. Hawlisch H. Monk P. N. Muller M. Klos A. Karp C. L. Kohl J. (2004) C5a mutants are potent antagonists of the C5a receptor (CD88) and of C5L2: Position 69 is the locus that determines agonism or antagonism. Journal of Biological Chemistry 279: 142–151. 14570896 10.1074/jbc.M310078200

[bibr33-1759091416687871] Pavlovski D. Thundyil J. Monk P. N. Wetsel R. A. Taylor S. M. Woodruff T. M. (2012) Generation of complement component C5a by ischemic neurons promotes neuronal apoptosis. FASEB Journal 26: 3680–3690. 22651932 10.1096/fj.11-202382

[bibr34-1759091416687871] Reichwald J. Danner S. Wiederhold K. H. Staufenbiel M. (2009) Expression of complement system components during aging and amyloid deposition in APP transgenic mice. Journal of Neuroinflammation 6: 35. 19917141 10.1186/1742-2094-6-35 PMC2784442

[bibr35-1759091416687871] Ricklin D. Hajishengallis G. Yang K. Lambris J. D. (2010) Complement: A key system for immune surveillance and homeostasis. Nature Immunology 11: 785–797. 20720586 10.1038/ni.1923 PMC2924908

[bibr36-1759091416687871] Rogers J. Cooper N. R. Webster S. Schultz J. McGeer P. L. Styren S. D. Civin W. H. Brachova L. Bradt B. Ward P. Lieberburg I. (1992) Complement activation by beta-amyloid in Alzheimer disease. Proceedings of the National Academy of Sciences 89: 10016–10020. 10.1073/pnas.89.21.10016 PMC50268 1438191

[bibr37-1759091416687871] Ryman D. Gao Y. Lamb B. T. (2008) Genetic loci modulating amyloid-beta levels in a mouse model of Alzheimer's disease. Neurobiology of Aging 29: 1190–1198. 17400334 10.1016/j.neurobiolaging.2007.02.017 PMC3745768

[bibr38-1759091416687871] Selkoe D. J. Hardy J. (2016) The amyloid hypothesis of Alzheimer's disease at 25 years. EMBO Molecular Medicine 8: 595–608. 27025652 10.15252/emmm.201606210 PMC4888851

[bibr39-1759091416687871] Sewell D. L. Nacewicz B. Liu F. Macvilay S. Erdei A. Lambris J. D. Sandor M. Fabry Z. (2004) Complement C3 and C5 play critical roles in traumatic brain cryoinjury: Blocking effects on neutrophil extravasation by C5a receptor antagonist. Journal of Neuroimmunology 155: 55–63. 15342196 10.1016/j.jneuroim.2004.06.003 PMC4766842

[bibr40-1759091416687871] Song W. C. (2012) Crosstalk between complement and toll-like receptors. Toxicologic Pathology 40: 174–182. 22109714 10.1177/0192623311428478

[bibr41-1759091416687871] Veerhuis R. (2011) Histological and direct evidence for the role of complement in the neuroinflammation of AD. Current Alzheimer Research 8: 34–58. 21143154 10.2174/156720511794604589

[bibr42-1759091416687871] Vergunst C. E. Gerlag D. M. Dinant H. Schulz L. Vinkenoog M. Smeets T. J. Sanders M. E. Reedquist K. A. Tak P. P. (2007) Blocking the receptor for C5a in patients with rheumatoid arthritis does not reduce synovial inflammation. Rheumatology (Oxford) 46: 1773–1778. 17965442 10.1093/rheumatology/kem222

[bibr43-1759091416687871] Wilcock D. M. Zhao Q. Morgan D. Gordon M. N. Everhart A. Wilson J. G. Lee J. E. Colton C. A. (2011) Diverse inflammatory responses in transgenic mouse models of Alzheimer's disease and the effect of immunotherapy on these responses. ASN Neuro 3: 249–258. 21995345 10.1042/AN20110018 PMC3227004

[bibr44-1759091416687871] Woodruff T. M. Ager R. R. Tenner A. J. Noakes P. G. Taylor S. M. (2010) The role of the complement system and the activation fragment C5a in the central nervous system. NeuroMolecular Medicine 12: 179–192. 19763906 10.1007/s12017-009-8085-y

[bibr45-1759091416687871] Woodruff T. M. Costantini K. J. Crane J. W. Atkin J. D. Monk P. N. Taylor S. M. Noakes P. G. (2008) The complement factor C5a contributes to pathology in a rat model of amyotrophic lateral sclerosis. Journal of Immunology 181: 8727–8734. 10.4049/jimmunol.181.12.8727 19050293

[bibr46-1759091416687871] Woodruff T. M. Crane J. W. Proctor L. M. Buller K. M. Shek A. B. de Vos K. Pollitt S. Williams H. M. Shiels I. A. Monk P. N. Taylor S. M. (2006) Therapeutic activity of C5a receptor antagonists in a rat model of neurodegeneration. FASEB Journal 20: 1407–1417. 16816116 10.1096/fj.05-5814com

[bibr47-1759091416687871] Woodruff T. M. Pollitt S. Proctor L. M. Stocks S. Z. Manthey H. D. Williams H. M. Mahadevan I. B. Shiels I. A. Taylor S. M. (2005) Increased potency of a novel complement factor 5a receptor antagonist in a rat model of inflammatory bowel disease. Journal of Pharmacology and Experimental Therapeutics 314: 811–817. 15879003 10.1124/jpet.105.086835

[bibr48-1759091416687871] Wyss-Coray T. Rogers J. (2012) Inflammation in Alzheimer disease-a brief review of the basic science and clinical literature. Cold Spring Harbor Perspectives in Medicine 2: a006346. 22315714 10.1101/cshperspect.a006346 PMC3253025

[bibr49-1759091416687871] Yao J. Harvath L. Gilbert D. L. Colton C. A. (1990) Chemotaxis by a CNS macrophage, the microglia. Journal of Neuroscience Research 27: 36–42. 2254955 10.1002/jnr.490270106

[bibr50-1759091416687871] Zhou J. Fonseca M. I. Pisalyaput K. Tenner A. J. (2008) Complement C3 and C4 expression in C1q sufficient and deficient mouse models of Alzheimer's disease. Journal of Neurochemistry 106: 2080–2092. 18624920 10.1111/j.1471-4159.2008.05558.x PMC2574638

