# Supplementary material for: C5a Increases the Injury to Primary Neurons Elicited by Fibrillar Amyloid Beta
Source: ASN Neuro. 2017 Jan 1;9(1):1759091416687871. doi: 10.1177/1759091416687871 (PMC5298486; doi:10.1177/1759091416687871)
Supplement: Supplementary material [file ASN687871_supplemental_figures.pdf]

Supplemental Figures for C5a Increases the Injury to Primary Neurons Elicited By Fibrillar Amyloid Beta

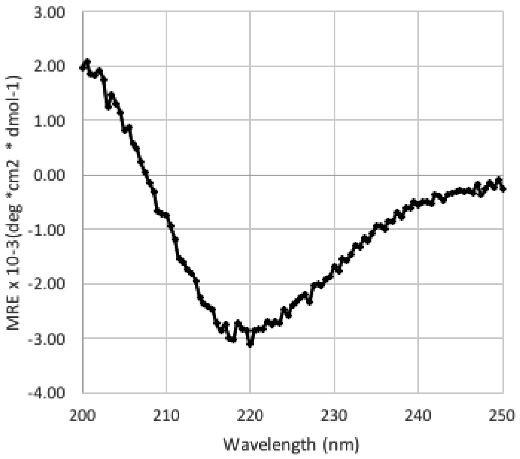

Supplemental Figure S1. Circular dichroism of amyloid beta preparation confirms beta sheet structure.

Amyloid beta peptide incubated for 20-24 hours shows minima at 218nm, indicative of β-sheet structure.

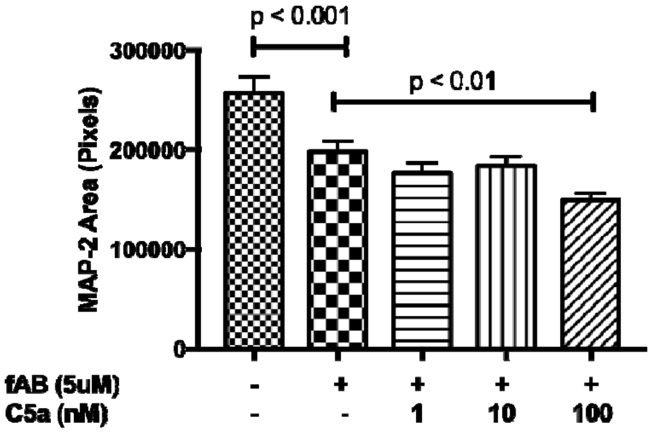

<u>Supplemental Figure S2</u>. C5a at a concentration of 100 nM increases MAP-2 loss in the presence of fAβ.

Primary neurons from WT mice were generated using E15-E16 pups and cultured for 7-10 days. The cells were then stimulated with 5 μM fAβ and 1, 10 or 100 nM hC5a for 24 hours. MAP-2 was visualized by immunocytochemistry (20x magnification) and quantified using ImageJ software as described in Materials and Methods. Data are presented as mean +/- SEM. n = 3 independent experiments, each with 3 coverslips per treatment, 3 images per coverslip. p values are calculated using One-way ANOVA, uncorrected Fisher's LSD test. Values of $p < 0.05$ were considered statistically significant.

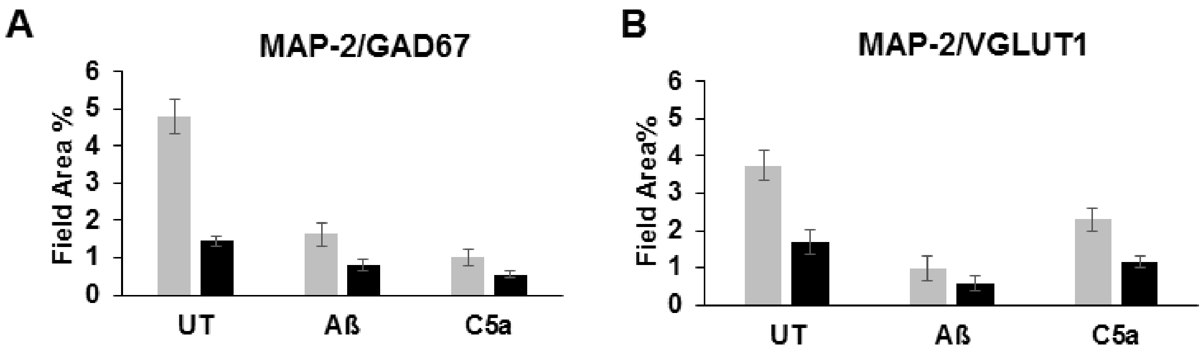

Supplemental Figure S3. fAβ and C5a kill both GABAergic and glutamatergic neurons in culture.

Quantification of the immunostaining of MAP-2 (grey bars) (A,B) and GAD67 (black bars) (A) or VGLUT1 (black bars) (B) in 7days cultures untreated or treated with 5 uM fAβ or C5a (100 uM). Bars represent the average of 5-7 images per coverslip (n=3) per condition +/- SEM.
